# Identifying the Incidence, Predictors, Outcomes, and Prevention of Stent Thrombosis (ST) in Post-percutaneous Coronary Revascularization Patients With Drug-Eluting Stents (DES): A Systematic Review

**DOI:** 10.7759/cureus.45150

**Published:** 2023-09-13

**Authors:** Mehvish Sohail, Priyansh Patel, Sidharth Midha, Surmai Shukla, Divyanshu Dhamija, Adedamola O Bello, Asma A Khan, Sai Dheeraj Gutlapalli, Sathish Venugopal

**Affiliations:** 1 Department of Internal Medicine, California Institute of Behavioral Neurosciences & Psychology, Fairfield, USA; 2 Department of Internal Medicine, Medical College Baroda, Baroda, IND; 3 Department of Radiology, Bharati Vidyapeeth University, Pune, IND; 4 Department of Radiology, California Institute of Behavioral Neurosciences & Psychology, Fairfield, USA; 5 Department of Internal Medicine, Qingdao University College of Medical Science, Qingdao, CHN; 6 Department of General Medicine, Government Medical College Amritsar, Amritsar, IND; 7 Department of General Medicine, California Institute of Behavioral Neurosciences & Psychology, Fairfield, USA; 8 Department of Internal Medicine, St. Martinus University, pontiac, USA; 9 Department of Internal Medicine, Richmond University Medical Center, Staten Island, USA; 10 Department of Neurology, California Institute of Behavioral Neurosciences & Psychology, Fairfield, USA

**Keywords:** stent thrombos, coronary artery disease (cad), dual-antiplatelet therapy (dapt), post pci, drug eluting stents (des)

## Abstract

Stent thrombosis (ST) is a rare but catastrophic event to happen to a stented coronary artery. The incidence of ST has greatly been reduced after the advent of modern drug-eluting stent (DES) implants, which have become the most preferred treatment option in the stenting category for coronary artery disease (CAD). Although the risk reduction by newer category implant provides substantial benefits, the possibility of thrombosis still exists mostly during the early stage of DES implantation. The development of ST after percutaneous coronary intervention (PCI) can be predicted by multiple factors, but advancements in early diagnostic techniques and modified stent types have greatly reduced the occurrence of this complication. Mortality, which is one of the complications of ST, is primarily influenced by patient-related factors such as incomplete treatment duration of dual antiplatelet therapy (DAPT). The duration of DAPT after DES implantation in patients with acute coronary syndrome (ACS) is determined based on individual characteristics, mainly considered in view of bleeding or ischemia risk. Risk evaluation systems like DAPT/precise-DAPT scores help tailor and personalize the duration of DAPT for each individual patient. This systematic review contains pertinent articles extracted from the PubMed database. We retrieved articles from various study categories, encompassing publications from the period spanning 2014 to 2022. Our analysis highlighted results from studies investigating different aspects contributing to ST development. The most favorable prevention option was the use of customized DAPT intervention based on patient-specific predictable factors. Several complications associated with ST were identified, including recurrent ST, major adverse cardiovascular events (MACE) encompassing all-cause mortality (including cardiac and non-cardiac mortality), cerebrovascular accidents (CVA) or transient ischemic attacks (TIA), hospitalization due to heart failure, and myocardial infarction requiring revascularization. Mortality was also observed as a significant outcome. The umbrella term of ST includes multiple causative factors. Although DES has improved patient survival rates vastly with its usage, careful risk factor assessment and required follow-up, in each individual being stented, further guarantee a more promising reduction in late adverse outcomes.

## Introduction and background

Stent thrombosis (ST) is a dreaded complication that can occur after percutaneous coronary intervention (PCI) [1]. Although PCI has proven to be life-changing in terms of increasing life expectancy by treating cardiac patients with stenosed coronary arteries [2], ST can occur occasionally as an unforeseen occurrence. However, it does not affect all patients who have undergone stent implantation, especially with the advent of drug-eluting stent (DES), which has led to revolutionary changes in prolonging life after PCI [2]. 

As the name implies, ST refers to the occlusion of a previously stented vessel that has now been blocked by a thrombus forming within the stent. This condition manifests as ST-elevation myocardial infarction (STEMI). DES is a widely used stent worldwide with a good safety profile. ST after DES implantation can be categorized into different types, based on the time of occurrence: acute ST (within 24 hours), sub-acute early ST (EST), within the first month, late ST (LST), between one month and one year, and very late ST (VLST) (after the first year) [1].

ST is not a commonly occurring event. A study reported an incidence of less than 5% among STEMI patients who underwent PCI with DES, implying that ST is not a very frequently encountered complication. It was determined that the sub-acute phase carries the highest risk for the development of ST [2]. The diagnosis and evaluation of ST necessitate a strong clinical suspicion. When any unexplained death occurs beyond 30 days after stent implantation, it is considered a possible diagnosis. An unexplained death or MI of the stented vessel without angiographic confirmation within 30 days of stent implantation is termed a probable diagnosis. However, a definitive confirmatory diagnosis can be established when ST is visualized on angiography and is clinically accompanied by acute ischemic symptoms at rest or new electrocardiogram (ECG) changes typical of ischemia [3,4].

A multitude of mechanisms can contribute to the pathology underlying the development of ST. These mechanisms include blood exposure to prothrombotic sub-endothelial constituents before re-endothelialization, the design and placement of stent struts, and the activation of the extrinsic pathway by polymer materials, which initiate coagulation. Similarly, slow blood flow creates a favorable environment for thrombus formation, specifically, it is the persistently slow-flowing coronary blood that activates the intrinsic coagulation pathway. After PCI, the most significant risk factor for ST development is inadequate pharmacological treatment, which is responsible for suppressing platelet reactivity due to, for instance, premature discontinuation of dual antiplatelet therapy (DAPT). Lastly, conditions associated with a systemic prothrombotic state e.g., acute coronary syndrome (ACS) or malignancy also increase the likelihood of ST development [4].

DES has also evolved over time. Second-generation DES have better mechanical performance and are more favored than first-generation DES because they offer a lower risk of LST and VLST particularly in high-risk patients while maintaining good efficacy in decreasing re-stenotic complications [5]. Biocompatible second-generation DES are highly effective, with durable polymers and coated drug formulations containing everolimus and zotarolimus. Both types have similar levels of efficacy compared to first-generation sirolimus-eluting stents (SES) but are more effective than paclitaxel-eluting stents (PES) from the latter class. However, only second-generation everolimus-eluting stents (EESs) significantly reduce the incidence of MI and ST, making them the safest type of DES [6].

ST can be caused by various mechanisms. Patient-related factors include advanced age, smoking, a higher body mass index (BMI), insulin-dependent diabetes, renal insufficiency, the extent of coronary artery disease (CAD), and a lower left ventricular ejection fraction (LVEF). Genetic variation in the CYP2C19 gene, which is involved in clopidogrel metabolism, predisposes carriers of this gene polymorphism to high on-treatment platelet reactivity (HTPR), enabling any possible early prediction of ST. Such ischemic events are influenced by other non-genetic factors like advanced age, diabetes mellitus, and higher BMI, which similarly contribute to HTPR to clopidogrel [7]. The variability in on-clopidogrel platelet reactivity has been documented as non-significant with 6-12% among the different genotypes, therefore, neither platelet function testing nor genetic testing for CYP2C19 polymorphism is recommended for tailoring DAPT unless in specific conditions where recurrent adverse effects require a change in treatment strategy. Hence, favoring the non-genotype-guided conventional clopidogrel therapy regimen in regular routine [8].

Lesion- and procedure-related factors identified by the intravascular ultrasound (IVUS) imaging technique indicate that the coronary anatomy complexities, such as lesions at a bifurcation or in the left main coronary artery (LMCA)/ left anterior descending (LAD), severely calcified lesions, residual lesions within 5mm of the stent, stent malposition, incomplete stent apposition, plaque protrusion, edge dissection, and residual stenosis, are associated with EST. Post-procedural factors include noncompliance with DAPT, with discontinuation within 30 days being the strongest predictor of EST [2,5]. Female gender has been indisputably found to be an independent factor in the development of most post-PCI complications in terms of in-hospital, 30-day, or one-year occurrence. However, men experienced more frequent in-hospital ST requiring urgent repeat PCI [9].

ST represents a potentially life-threatening outcome following PCI and is associated with a mortality rate of 5-10% during the initial presentation within the in-hospital stay and 10-25% after 30 days. The complication of recurrent stent thrombosis (rST) ranges from 15-20% at five years, with a three-year mortality rate related to rST, accounting for 30% [10]. DAPT, which includes aspirin plus P2Y12 inhibitors, is the most effective approach to reducing the risk of ST. Potent third-generation P2Y12 inhibitors such as prasugrel, ticagrelor, and cangrelor have been shown to be more efficacious in protecting against ST compared to clopidogrel in patients with non-ST elevation myocardial infarction (NSTEMI) and STEMI. These inhibitors act earlier than clopidogrel and provide greater platelet inhibitory activity [11]. However, consideration needs to be made as clopidogrel remains the most widely used and approved drug for patients undergoing elective PCI for stable angina. Patients with stable angina are recommended to undergo DAPT for six months following PCI. In patients with ACS, a 12-month treatment duration is advised, but in cases of a high risk of bleeding, a shorter duration of six months can be prescribed. Similarly, ACS patients with multivessel coronary disease could benefit from prolonged DAPT if a high risk of ischemia is a concern. Therefore, it is necessary to conduct an individual assessment of both ischemia and bleeding risk utilizing risk assessment systems, such as the PRECISE-DAPT score. This is especially helpful for patients implanted with DES, as they have more flexibility in terms of premature discontinuation of DAPT based on their risk profile [3,8].

## Review

Methods

Literature Search

We conducted a systematic literature search to include pertinent articles retrieved through a selective search in the National Institute of Health-NIH (PubMed, PubMed Central, Medline). Only full-text articles related to the topic were extracted from the databases. Articles published between 2014 and 2022 were selected. We searched for randomized controlled trials, clinical trials, metanalysis, systematic reviews, and reviews. Reports collected to formulate this systematic review were based on Preferred Reporting Items for Systematic Reviews and Meta-Analysis (PRISMA) 2020 guidelines [12]. A medical subject headings (MeSH) and keyword strategy were used to search the database, including articles with information related to stent thrombosis, coronary intervention, and drug-eluting stents. Eligibility criteria included articles published in the English language only. Papers discussing the development of ST in selected populations presenting with stable angina or ACS and undergoing PCI stenting via DES were included. We conducted a search for papers focusing on the identification of factors related to the prediction, recurrence, incidence, outcomes, treatments, prevention, and imaging techniques associated with the development of ST. Articles that mentioned prophylactic treatment of ST with antiplatelet therapy given for variable durations and their effects on the outcomes of ST were considered for inclusion in our review article. Articles that mention different DES types were reviewed with a brief addition to the systematic review. The exclusion criteria encompassed studies that compared outcomes between PCI and coronary artery bypass graft (CABG); studies focusing on ST in calcified vessels; studies examining outcome management in ST patients with arrhythmias, and studies discussing outcomes related to major adverse cardiovascular events (MACE). Comparative studies between drug-coated balloons (DCB) and DES were not included in this study. Gray literature containing unpublished reports or non-commercially available literature and proposals were not considered part of this review. Table 1 demonstrates the population, intervention, comparison, and outcomes (PICO) criteria as the primary framework for determining the eligibility of selected papers to be included in this systematic review.

**Table 1:** PICO criteria used for inclusion framework ST: stent thrombosis; DES: drug-eluting stent; PCI: percutaneous coronary intervention; ARC: academic research consortium; CABG: coronary artery bypass graft; BMS: bare metal stent

PICO constituents	Groups included in the systematic review
Population	Patient population presenting with ST defined according to ARC classification
Race	All races
Gender	Males and females
Age	45 years old and above
Intervention	PCI with DES
Comparison	No comparative groups were studied in terms of medical treatment vs. PCI vs. CABG. Similarly, no papers including different stent types with BMS or DES were specifically extracted.
Outcome	ST

Results

**Figure 1:**
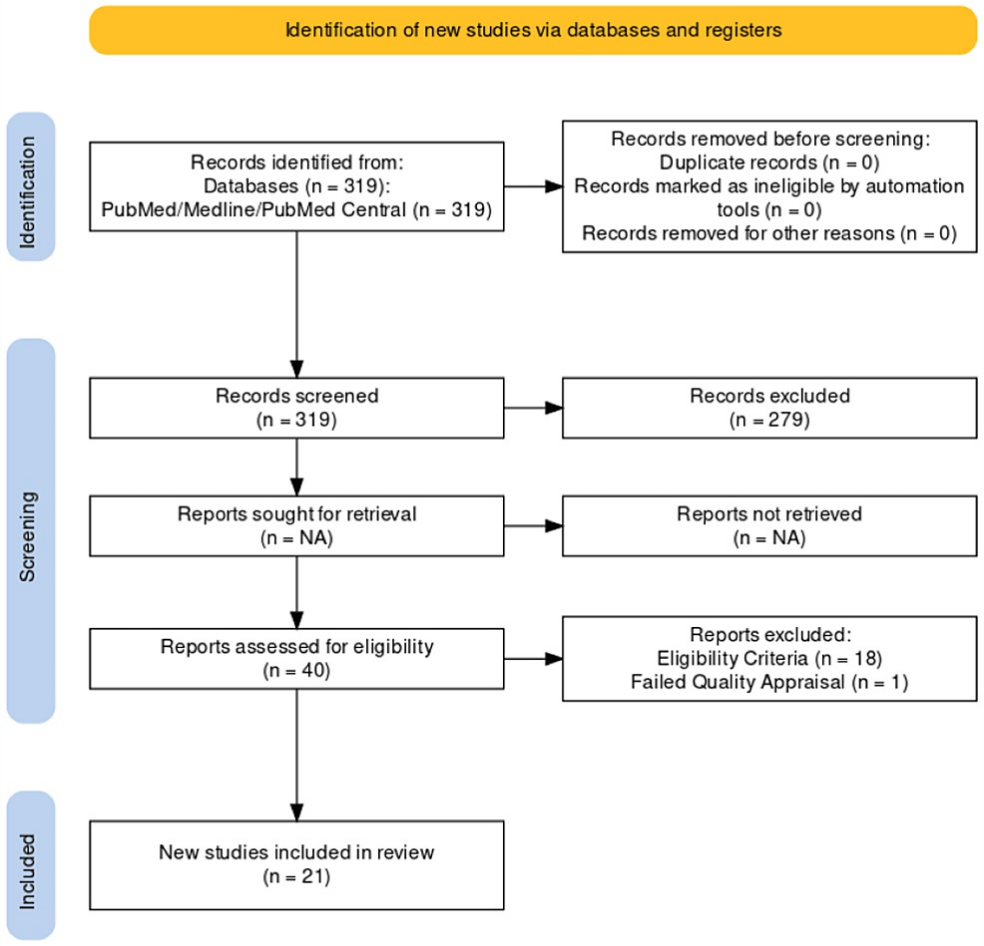
PRISMA (Preferred Reporting Items for Systematic Reviews and Meta-Analysis) flowchart

All relevant articles selected for inclusion in our study were thoroughly checked and screened for any duplicate articles. All co-authors contributed to the shortlisting of the reference articles. Articles were further evaluated through the availability of their full text, and articles that justified the inclusion and exclusion criteria were chosen for addition to our study. After screening articles by title and abstract total of 21 articles were included and assessed for retrieval. Studies from different populations were added, including those from American, European, and Asian literature. Figure 1 shows a PRISMA flow diagram representing the search strategies used for our article. 

Table 2 mentions the studies included and discusses various aspects of our systematic review, focusing on different variables involved in ST. There have been mentioned various determinants in the literature to identify the cause of ST. With the utilization of advanced DES in current practice, there has been a considerable reduction in the occurrence of ST. Additionally, post-procedure DAPT has emerged as the most crucial factor in minimizing the rate of complications associated with ST. DAPT can be easily adjusted according to individual factors.

**Table 2:** Characteristics of included articles. PCI=percutaneous coronary intervention, DES=drug-eluting stent, RCT=randomized control trial, ST=stent thrombosis, MI=myocardial infarction, DAPT=dual anti-platelet therapy, CAD=coronary artery disease, LVEDP=left ventricular end-diastolic pressure, TIMI flow grade=thrombolysis in myocardial infarction (TIMI coronary grade flow is a grading system of coronary reperfusion), PPI=proton pump inhibitors, MACE=major adverse cardiovascular event, ISA=incomplete stent apposition, RST=recurrent stent thrombosis, CYP2C19∗2 or CYP2C19∗3=member of the cytochrome P450 gene family, HTPR=high on-treatment platelet reactivity, EES=everolimus-eluting stent, BES=biolimus-eluting stents, CK=creatine kinase, OCT= optical coherence tomography, LOF=loss of function.

Author name	Publication year	Study type	Aim of study	Outcomes/result	Follow-up period
Kuramitsu S et al. [1]	2019	REAL-ST (Retrospective Multicenter Registry study of ST.	Risk factors of definite ST after second-generation DES implantation; long-term clinical outcomes of patients with definite ST	Risk factors vary according to the time of ST presentation; definite ST patients have unfavorable long-term outcomes	Four years
Kumar R et al. [2]	2022	Prospective observational study	Acute and sub-acute ST incidence, predictors, and outcomes	Male gender, LVEDP ≥ 20 mmHg, and pre-procedure TIMI flow grade were independent predictors of acute or sub-acute ST	Six months
Ullrich H et al. [3]	2020	Literature search	Predictors and prevention of coronary ST	Multiple patient and procedure-related factors can be involved in ST development DAPT and early diagnostic imaging techniques can reduce the incidence of ST	N/A
Kuramitsu S et al. [5]	2021	Literature review	Pathophysiology, risk factors, and outcome of ST after DES use.	Multiple factors contribute to ST development with unfavorable prognosis of mortality and recurrent ST to a greater extent in high-risk patients	N/A
Navarese E P et al. [6]	2014	Meta-analysis of RCT	Comparison between first- and second-generation DES	Second-generation EES has the least risk of ST and MI	N/A
Ma Q et al. [7]	2019	Prospective observational	Role of platelet reactivity to clopidogrel in genetically susceptible patients presenting with acute vascular event	Older age, raised BMI, regular use of DAPT, diabetes, and polymorphic variants with LOF alleles in CYP2C19^∗^2 or CYP2C19^∗^3 carriers are significantly associated with HTPR to clopidogrel	29 months
Kim HL et al. [9]	2019	Observational study	Gender influence on the in-hospital outcome after PCI with DES	Males had significantly more ST and urgent repeat PCI than females who dominantly had other composite events	N/A
Armstrong E J et al. [10]	2014	Multicenter registry analysis from 2005-2013	Identify incidence, predictors, and outcomes of rST.	rST is a more common complication in older patients and has a higher chance of mortality than initial ST	3.3 years
White H et al. [11]	2015	RCT	Comparison between cangrelor and clopidogrel use, who received bivalirudin during PCI	Bivalirudin and cangrelor together function to reduce MI and acute ST along with reduced bleeding complications compared to clopidogrel	N/A
Kubo T et al. [13]	2014	RCT	The vascular response between biodegradable BES polymer vs. durable EES polymer using OCT as an imaging modality	ST favoring vascular responses including neointima proliferation and malapposed/under-covered struts, were reported less with EES use	8-12 months
Cavallari L et al. [14]	2018	Multicenter pragmatic investigation	CYP2C19 genotype affecting outcomes after PCI by determining anti-platelet therapy	Higher rate of ST in the LOF-clopidogrel group	12 months
Kosmidou I et al. [15]	2020	Metanalysis of RCT	Five years outcome difference between males and females after PCI	DES-implanted women had a similar rate of ST at 30 days and at five years with that of men but had more other composite events	Five years
Ishihara T et al. [16]	2022	Multicenter retrospective observational study	Identify predetermining factors contributing to long-term mortality after ST development.	Hemodialysis, high peak CK levels, and lesion in the left main coronary independently contributes to 10-year mortality after ST development	N/A
Attizzani G et al. [17]	2014	Review article	Mechanism and clinical relevance of incomplete stent apposition	ISA contributes to ST development likely by inducing prothrombotic vascular changes, requiring adequate stent information related to vascular parameters.	Studies with a follow-up period of six to nine months
Basaraba J et al. [18]	2016	Systematic review	DAPT durations after PCI with DES	The short duration of DAPT of three to six months had equal efficacy in the prevention of ST as 12 months DAPT in low-risk populations but warrants judicious application while considering high-risk patients.	Studies with follow-up duration of 12 months.
Valgimigli M et al. [8]	2017	Practice guideline article	DAPT guidelines for usage in CAD patients	-	-
Didier R et al. [19]	2017	RCT	Comparing DAPT with six months to that of 24 months in an aspirin-sensitive population, after DES implantation	Non-inferiority between both DAPT groups in terms of bleeding and ST	Two years
Piccolo R et al. [20]	2017	Systematic review	To identify early (within 90 days) adverse outcomes occurring after clopidogrel discontinuation studied in short-term and long-term DAPT groups, after DES implantation	ST and MI risk were non-significant in both study groups but early MACE was documented higher in the group discontinuing clopidogrel after long-term use	Three subgroup time stages were being followed-up i.e., 90 days before intervention, zero to 90 days, and 91 to 180 days after DAPT discontinuity
Bundhun P K et al. [21]	2017	Systematic review	Association of concomitant use of PPI and clopidogrel after PCI and their related adverse outcomes	Significantly higher adverse events of MACE, MI, and ST following PCI, especially in omeprazole users	Short-term (less than one year) and long-term follow-up (more than a year) up til three years
Luo L et al. [22]	2019	Metanalysis	Comparison of monotherapy of P2Y12 inhibitor with DAPT after PCI	P2Y12 inhibitor monotherapy has favorable outcomes in the prevention of ischemic and bleeding events	Median follow-up period of 12 months

Quality Appraisal of Studies

We analyzed all shortlisted articles for quality appraisal and exclusion of potential risk of bias. Table 3 represents the Newcastle-Ottawa Scale (NOS) for assessing the quality of included cohort studies.

**Table 3:** Summary of the Newcastle-Ottawa Scale for cohort studies Good quality=3 or 4 (Y) in the selection domain AND 1 or 2 (Y) in the comparability domain AND 2 or 3 (Y) in the outcome domain Fair quality=2 (Y) in the selection domain AND 1 or 2 (Y) in the comparability domain AND 2 or 3 (Y) in the outcome domain Poor quality=0 or 1 (Y) in the selection domain AND 0 (Y) in the comparability domain AND 0 or 1 (Y) in the outcome domain Y, yes; PY, partial yes Adapted from Kuramitsu et al. [1] and Ullrich et al. [3]

First author	Selection				Comparability	Outcomes			Total	
	Representativeness of exposed cohort	Selection of non-exposed cohort	Ascertainment of exposure	Outcomes not present at the start of the study		Assessment of outcome	Sufficient follow-up	Adequacy of follow-up		
Kuramitsu S et al. [1]	Y	Y	Y	Y	Y	Y	Y	Y	8	Good
Cavallari L et al. [14]	Y	Y	Y	Y	Y	Y	Y	Y	8	Good

The Scale for Assessment of Narrative Review Articles (SANRA Checklist) was used for the quality appraisal of narrative reviews, as mentioned in Table 4.

**Table 4:** SANRA Checklist for Review Articles SANRA=Scale for Assessment of Narrative Reviews Articles score of 2=high standard; score of 1=intermediate standard; score of 0=low standard

First author	Importance	Aim of study	Literature search	Referring and presentation	Evidence level	Endpoint data presentation	SANRA standard
Kumar R et al. [2]	2	2	2	2	1	2	Good
Ullrich H et al. [3]	2	2	2	2	2	2	Good
Claessen B E et al. [4]	2	1	2	2	2	2	Good
Ma Q et al. [7]	2	2	1	1	1	2	Moderate
Ishihara T et al. [16]	2	2	0	2	2	2	Good

 A randomized control trial was assessed using the Cochrane bias assessment tool, demonstrated in Table 5.

**Table 5:** Assessment of clinical trials using the revised Cochrane risk-of-bias tool for randomized trials (RoB 2)

Clinical trial author's name	Random sequence generation (selection bias)	Intervention nonadherence	Incomplete outcome data (attrition bias)	Blinding of outcome assessment (detection bias)	Selective reporting (reporting bias)
White H [11]	low risk	low risk	low risk	low risk	low risk
Didier R [19]	low risk	low risk	low risk	unclear	low risk

Table 6 assessed systematic reviews and meta-analyses using the assessment of multiple systematic reviews (AMSTAR 2) tool. Results of quality appraisal with respect to risk of bias were reported as evoking low, moderate, or high risk.

**Table 6:** ARMSTAR checklist for review articles ROB: risk of bias Low risk of bias >70% points; moderate risk of bias=50-70% points; high risk of bias <50% points

First author	PICO framework included	Pre-defined methods and research proposal	Design of study outlined	Thorough literature search	Selection of studies by two or more individuals	Extraction of data by two or more individuals	Record and reasons for reports excluded	Detailed description of included studies	Adequate RoB procedure followed	Disclosure of funding sources	Appropriate statistical analysis	Effect of RoB of primary studies on meta-analysis results	RoB considered in primary studies	Investigation of heterogeneity	Small study bias	Potential conflicts reported	Total score (/16)	Final quality appraisal of the review
Luo L [22]	Y	N	Y	PY	Y	Y	N	Y	Y	N	Y	Y	Y	Y	Y	Y	12.5	High
Navarese E P [6]	Y	Y	Y	Y	Y	Y	Y	Y	Y	Y	Y	N	N	Y	Y	Y	14	High
Basaraba J [18]	Y	Y	Y	Y	Y	Y	Y	Y	PY	Y	Y	Y	Y	Y	Y	Y	15	High
Piccolo R [20]	Y	Y	Y	Y	Y	Y	Y	Y	Y	Y	Y	Y	Y	Y	Y	Y	16	High
BundhunP K [21]	Y	Y	Y	Y	Y	Y	Y	Y	N	Y	Y	N	N	Y	Y	Y	13	High

Discussion

Introduction

PCI is the most frequently used treatment modality for angiographic studies and interventions related to CAD patients. Stents used during the procedure help maintain the patency of stenosed arteries and reduce the risk of spontaneous closure. Stenting prevents restenosis by interfering with the remodeling of arterial architecture that promotes neointimal proliferation favoring ST. ST can present variably as chest pain with ischemic changes in the ECG or, in severe cases, result in death. The most advanced DES used efficiently with DAPT, contributes the least to this complication compared to bare metal stents (BMS) and other techniques using low-pressure balloon inflation and single antiplatelet therapy. DES works by releasing antiproliferative agents in a controlled manner through a less toxic, more biocompatible polymer with thinner stent struts made of modern alloys. Among the DES, EES has been found to have the lowest chance of ST at 0.7% (p = 0.002) [3].

Classification

The classification of ST is made on the basis of the Academic Research Consortium (ARC) definition. It defines ST in terms of time elapsed during presentation as well as the certainty level, according to different underlying pathogenesis and clinical presentation [3]. Table 7 represents the ARC classification of ST [4].

**Table 7:** Academic Research Consortium (ARC) classification of ST ST: stent thrombosis, MI: myocardial infarction, PCI: percutaneous coronary intervention Adapted from Claessen et al. [4]

Level of Certainty	Timing
Definite	Early
Angiographic or pathological confirmation of partial or total thrombotic occlusion within the peri-stent region	Acute (<24 hours)
AND at least one of the following additional criteria:	Subacute (24 hours to 30 days)
Acute ischemic symptoms	
Ischemic electrocardiogram changes	
Elevated cardiac biomarkers	
Probable	Late
Any unexplained death <30 days of stent implantation	31 days to 1 year
Any myocardial infarction related to documented acute ischemia in the territory of the implanted stent without angiographic confirmation of stent thrombosis and in the absence of any other obvious cause	
Possible	Very late
Any unexplained death beyond 30 days	>1 year

Pathophysiology

The pathophysiology of ST is multifactorial. Patient and lesion-related factors constitute the most critical determinants of ST, as they are usually implicated in the earliest phase of development. The vascular inflammatory response, likely attributable to polymer-induced hypersensitivity, plays a significant role in platelet reactivity and endothelialization which further contributes to neointimal coverage. These changes ultimately ascertain the extent of ST developing in the later stages, manifesting as LST or VLST. Therefore, it is generally challenging to identify VLST [4]. The vascular healing response differs between stents with durable polymers and those with biodegradable polymers. The NEXT trial, conducted for a duration of 8-12 months, followed two study populations: one with EES using a durable polymer and the other with BES using a biodegradable polymer. Using optical coherence tomography (OCT) as an imaging marker, the trial concluded that EES had a more favorable outcome in preventing ST, with a lower incidence of contributing factors such as neointimal growth and malapposed struts, along with a decreased frequency of uncovered struts (4±5% vs. 11±13%, p=0.016). EES with flexible stent designs, biocompatible polymers, and thin stent struts promote early and adequate neointimal coverage, reducing the risk of ST development. Incomplete neointimal coverage can lead to the formation of neoatherosclerosis, which may rupture and present as VLST (after the first year). Although the bio-absorbability of BES presumably offers less hypersensitivity risk, further validation is required to prove its benefits in preventing VLST [13].

Predictors

Table 8 summarizes the Risk factors for ST development after second-generation DES implantation [3-5].

**Table 8:** Contributing predictors in the development of stent thrombosis STEMI=ST elevation myocardial infarction, LAD=left anterior descending artery, ISR=in-stent restenosis, des=drug-eluting stents, BMS=bare metal stents, ACS=acute coronary syndromes, VLST=very late stent thrombosis, DAPT=dual anti-platelet therapy, LVEF=left ventricular ejection fraction

	Early ST [3-5]	Late ST [3-5]	(Very) Late ST [3-5]
Patient characteristics	STEMI, heart failure, peripheral artery disease, diabetes mellitus, ACS, nonadherence to DAPT, genetic polymorphisms, malignancy, thrombocytosis	Malignancy (p <0.0001), diabetes mellitus (p = 0.0093), hemodialysis, reduced left ventricular function, young age	End-stage renal disease, smoking, STEMI, LVEF<40%, nonadherence to DAPT (unknown for very late ST)
Lesion	Bifurcation lesion, LAD, vessel size, lesion length, thrombus, saphenous vein grafts.	severely calcified lesion, LAD lesion, and vein graft stenting.	LAD lesion, incomplete endothelialization, ISR, delayed healing, previous brachytherapy, vein graft stenting
Procedural	Stent under-sizing, stent under-expansion, stent malapposition, stent dissection, no pre-procedural thienopyridine administration, stent length, residual lesion within 5mm stent range.A stent diameter of <3.0mm	Bifurcation lesion treated with 2 DES stent, Overlapping stent	DES (compared with BMS), permanent polymer DES (compared with bioresorbable polymer DES), overlapping DES
Post-procedural	Discontinuation of antiplatelet therapy	Discontinuation of antiplatelet therapy	Discontinuation of antiplatelet therapy (unknown for VLST), late acquired stent malapposition

The incidence of ST in up to one-year follow-up ranges from 0.6% to 3.4% with DES [4]. A myriad of factors can predict ST development. Listed in Table 9 are some significant patient-related demographic multivariant and univariant factors that contribute to the formation of ST from a study by Kumar et al. [2].

**Table 9:** Patient-related factors for ST development TIMI flow grade=thrombolysis in myocardial infarction, EST=early stent thrombosis, LST=late stent thrombosis, ISR=in stent restenosis Adapted from Kumar et al. [2]

Patient-related risk factors for ST development			
Study population (1756)	Odd’s Ratio (95% CI)	Multivariable factors	ST 86(4.9%)
Male	2.51 (1.21–5.2)	0.013	N/A
LV end-diastolic pressure ≥ 20 mmHg	2.55 (1.31–4.98)	0.006	N/A
Pre TIMI flow grade 0	3.27 (1.61–6.65)	0.001	N/A

In the same study, significant p-values were obtained in the univariate logistic regression analysis for the following parameters: Older age, neutrophil count, intubation, cardiac arrest, hypertension, smoking, diabetes, use of an intra-aortic balloon pump, intra-procedure slow-flow/no-reflow, final sub-optimal TIMI flow (<3), arrhythmias, bleeding, cardiogenic shock, and Killip class III/IV. Notably, diabetes and kidney failure emerged as strong independent predictors of sub-acute and LST [2]. Malignancy is also significantly associated with LST [3]. However, small vessel disease and ISR are considered strongly linked with EST [2].

By considering genetic variability, interventions aimed at preventing ST can lead to a reduction of 6-12% in susceptible populations through the personalized selection of DAPT. Patients with CYP2C19 loss-of-function (LOF) alleles have low or absent CYP2C19 activity, predisposing them to ST as they cannot metabolize clopidogrel effectively. Therefore, in the post-PCI phase, CYP2C19-guided antiplatelet therapy is optimized with alternative P2Y12 inhibitor antiplatelet therapy, such as prasugrel and ticagrelor. However, due to the expense and risk of bleeding associated with these alternate drugs, their use is limited to patients with high-risk anatomy or diabetic complications. ST is generally highest during the initial 30-day period but may extend up to three years. In this context, the CYP2C19 genotype plays a major role in guiding therapy regarding when to switch to the cheaper alternative. However, CYP2C19 genetic testing is not routine practice and is limited to high-risk heart failure Killip class IIb patients [14].

The incidence of ST is higher in males (p-value = 0.003) in the in-hospital setting, requiring urgent hospitalization (p-value=0.015) within six hours to 24 hours [9]. However, there is no significant difference between both sexes in the first year and onwards from one to five years. Women have an increased rate of other composite outcomes, including non-stent-related complications from MI, MACE, and TLR (target lesion revascularization) after one year [15].

Procedure-related intracoronary imaging-guided PCI via IVUS and OCT has a lower frequency of ST compared to angiographic-guided procedures (0.6% vs. 1.2%, p = 0.005). High strut thickness also contributes to ST by increasing shear stress, platelet activation, and reducing antiproliferative drug delivery. The SORT OUT VII study compared SES (60-µm stent strut thickness) with BES (120-µm stent strut thickness) and favored thinner struts (0.4% vs. 1.2%, p = 0.034) in reducing the chances of definite ST. According to the prospective PESTO registry, strut mal-apposition accounts for 48% of early ST and 31% of LST, as it creates a low-flow area that promotes platelet activation. Stent under-expansion causes EST by 26% [3].

Early discontinuation of DAPT accounts for most cases of subacute ST and LST [3]. DAPT is recommended for six months in patients with stable angina following PCI, with exceptions made if there is a high risk of fatal bleeding - e.g. previous bleeding episode, bleeding diatheses [18] - which may require dose alteration after one to three months. For ACS patients, DAPT is continued for 12 months, with the exception of cases with a risk of fatal bleeding, where therapy duration is reduced to six months [3]. Similarly, an extended duration of DAPT for more than 12 months can be given to patients with complex coronary lesions or those with multi-vessel coronary disease following ACS. Individual risk stratification for ischemia and bleeding needs to be considered when deciding the duration of DAPT therapy, such as using the PRECISE-DAPT score [3].

rST, which occurs after the initial ST, is also a concerning outcome with a hazard ratio of 16% in the first year and 24% in the fifth year. Age (median 64 years), an initial bifurcation lesion, and a larger proximal reference vessel diameter are independent predictors of rST. Female patients and non-hypertensive patients are more likely to experience rST. In addition to DAPT, these patients can be considered for additional antithrombotic therapy with rivaroxaban to reduce recurrent ischemia, although the risk of bleeding should always be taken into account [10].

OCT and IVUS are imaging techniques used to visualize stent-related pathologies that contribute to the development of ST. They assist in identifying malposition, uncovered stent struts, evagination, in-stent restenosis (ISR), and no atherosclerosis, which can be treated by prolonging DAPT and using the appropriate generation of DES [3]. Subsequently, it can be said that minimal residual stenosis, absence of dissection, exclusion of stent under-sizing, and appropriate stent apposition with sub-optimal final coronary flow and no significant complications like MACE, frames a successful PCI [4]. OCT has a 97% accuracy in determining morphological abnormalities of stents, such as mal-apposition and under-expansion [3]. Stent malapposition refers to the condition when stent struts do not make contact with the vessel wall, with incomplete neointimal coverage favoring the development of EST (48%) and LST (31%). Appropriate sizing and use of imaging techniques to confirm proper stent expansion can prevent these complications with post-dilation of the stent and prolonged DAPT. DCB can be an alternative if neo-intimal hyperplasia with malapposition exists [3-5]. On the other hand, stent under-expansion is an inadequately expanded stent compared with the adjacent normal reference segment and is a highly influential predictor of TLR [4]. Stent under-expansion accounts for 26% EST [3] and occurs due to rigid calcified arterial lumen restricting stent expansion; high-pressure balloon expansion can be attempted in these circumstances [4]. Incomplete stent apposition (ISA), which refers to the lack of contact between stent struts and the vessel wall, is commonly seen after DES implantation and is a major cause of LST and VLST development (73.9%). DES is considered an independent predictor of ISA (odds ratio: 9.8, 95% confidence interval: 2.4 to 40.4; p = 0.002). Several pathological mechanisms contribute to the development of ISA, including inadequate stent size compared to luminal dimensions, ACS, bifurcation lesions, vessel tapering, and chronic total occlusion of coronary vessels, resulting in smaller sizes due to occlusion-induced hypo-perfusion. IVUS and OCT can also be used to diagnose ISA. These imaging methods enable adequate stent sizing, including the selection of optimal stent length, while also providing high-resolution cross-sectional images for diagnosis. IVUS has a high resolution of 150 μm compared to OCT, which uses infrared light to detect lesions with approximately ten-fold higher axial resolution (approximately 10- to 15-μm axial resolution) [17].

Along with its diagnostic value, IVUS can be used as an interventional tool to guide DES implantation and has been associated with a lower risk of overall mortality (OR: 0.64; (0.51; 0.81); p <0.001), reduced rates of MI (OR: 0.57; (0.42; 0.78), p <0.001), and ST (OR: 0.59; (0.42; 0.82); p = 0.002) [3]. IVUS-guided stent studies have shown particular benefits for patients presenting with ACS and complex lesions, mainly in reducing the occurrence of EST [1]. Studies such as CLI-OPCI, OPINION, and ILUMIEN III suggest that OCT use during PCI also improves the clinical outcome of patients and is not found to be inferior to IVUS. Although novel OCT-based techniques for guiding coronary stent implantation have shown similar favorable outcomes, large-scale clinical trials are needed to further justify their equal efficacy [3].

ST is a widely discussed complication, primarily due to its impact on mortality. First-generation DES, second-generation DES, and third-generation DES accounted for 19.3%, 36.9%, and 6.4% of ST cases, respectively. A retrospective study using data from a multicenter registry provided insights into long-term outcomes associated with DES thrombosis [16]. Table 10 outlines the outcomes of DES thrombosis [16,10].

**Table 10:** Outcomes of ST LMT: Left main trunk;  rST: recurrent ST; EST: early stent thrombosis; LST: late stent thrombosis; VLST: very late stent thrombosis; MACE: major adverse cardiovascular events; ST: stent thrombosis

Outcomes of ST	Risk of occurrence	Associated factors related to the outcomes	Time period affecting the outcomes of ST
Mortality [16]	10‐year cumulative mortality after ST was 33.8%.	Patients with ST having Left main trunk (LMT) lesions had the highest mortality while those with lesions in the right coronary artery had the lowest mortality. Independent predictors for all‐cause death were hemodialysis, culprit lesions in the LMT and left coronary artery, and peak CK.	Mortality in LST was higher than that found in patients with EST and VLST.
	5‐year mortality was 24.4 - 39.0% after ST occurrence.		
	Cardiac death - 14.7%		
	Non-cardiac death - 7.3%		
Recurrent ST [10]	1 year risk-11-16%	Likely in hypertensive (p-value - 0.005). Recurrence was observed more in patients having higher peak CK at initial ST (p-value - 0.05)	3-year MACE after rST is more significant than with initial ST.
	5 year risk-20-24%		

DAPT is the most intensively investigated treatment modality to reduce post-procedural ST complications. It consists of a combination of aspirin with an oral inhibitor of the platelet P2Y12 receptor for adenosine 5'-diphosphate (ADP). Given the high risk of LST with DES, the current standard of practice requires a DAPT duration of 12 months, especially in ACS patients undergoing PCI [18]. Studies favoring DAPT prolongation beyond 12 months after MI or after PCI suggest the potential advantage of reduced future spontaneous MI risk which is otherwise associated with a 15% mortality rate in these patients [8].

The PEGASUS-TIMI 54 trial conducted in a patient population with prior MI, and the PRODIGY trial with a majority of ACS patients compared short and extended DAPT to conclude a favorable reduction in ischemic event risk, but also found a significantly higher incidence of bleeding in the 24-month DAPT group compared to the six-month group [19].

DAPT duration continues to evolve with different considerations with respect to patient history. The ITALIC trial gathered data comparing the ischemic and major bleeding risk at 12 months in patients undergoing PCI with second-generation DES. The results showed the non-inferiority of DAPT between six months vs. one year, with an absolute risk difference of 0.11% (p-value = 0.0002), and between six months vs. two years, with an absolute risk difference of 0.22% (p = 0.0197). The composite endpoints (e.g., all-cause mortality, major bleeding, stroke, MI) were also not significant, occurring in 3.5% of the six-month group and 3.7% of the 24-month group (p = 0.79). There was a nonsignificant numerical increase in the mortality rate with longer DAPT (2.2% vs. 1.2%; p = 0.11). Patients over 75 years old had more composite events with 24 months of DAPT. The ITALIC trial also supports the most recent European and updated American guidelines for DAPT after PCI, especially considering newer generation DES, which recommend continued reduced six months DAPT in the non-ACS group and three months DAPT in patients at risk for bleeding. Patients at high risk for ischemia should be kept on 12 months of DAPT, as otherwise, the risk of ST is doubled. On the other hand, the DAPT study reported not only an increase in hemorrhagic events, as expected but also significantly higher mortality with extended DAPT [19]. Table 11 provides a summary of the relevant studies mentioned above, including different DAPT durations and their related outcomes [19].

**Table 11:** Variable DAPT durations and its outcomes. PEGASUS-TIMI 54=Prevention of Cardiovascular Events in Patients With Prior Heart Attack Using Ticagrelor Compared to Placebo on a Background of Aspirin–Thrombolysis in Myocardial Infarction 54, PRODIGY=Prolonging Dual-Antiplatelet Treatment After Grading Stent-Induced Intimal Hyperplasia Study, ITALIC=Is There A Life for DES after discontinuation of Clopidogrel, ACS=acute coronary syndrome, CAD=coronary artery disease, PCI=percutaneous coronary intervention, DAPT=dual anti-platelet therapy, MI=myocardial infarction, EES=everolimus-eluting stent, PES=paclitaxel-Eluting Stent, ST=stent thrombosis, MACE=major adverse cardiovascular event

Trials conducted to study DAPT duration	Duration of DAPT used in the trials	Outcomes related to different durations of DAPT
PEGASUS-TIMI 54 trial in patient population with prior MI/PCI (Only study to allow re-introduction of DAPT after period of discontinuation to combat ischemic events) [19]	Compared six months and 24 months DAPT(studying specifically used ticagrelor)	Higher major bleeding risk in 24 months group using DAPT, but reduced ischemic events
PRODIGY trial with majority ACS and remaining stable CAD population and various different stent types tested [19]	Compared six months and 24 months DAPT	Higher major bleeding risk in 24 months group using DAPT, especially in CAD patients. PES only showed reduced ST with extended DAPT contrary to EES favoring shorter DAPT for ST, MACE reduction.
ITALIC trial [19]	Compared six months DAPT to 12 or 24 months DAPT	No difference in ischemic event reduction and major bleeding
DAPT study [19]	Patients treated with prasugrel within 12 months after PCI randomly allocated to stop drugs or allowed to continue till 30 months	Higher mortality and fatality along with moderate/major bleeding with extended DAPT but reduced ST (0.4% vs. 1.4%; p-value < 0.001) and MACE.

In some circumstances, such as urgent surgical procedures or episodes of major bleeding, the duration of DAPT can be reduced from the standard 12 months to a three or six-month period. Studies have shown that there is no statistically significant difference in the increased risk of ST with shorter-term DAPT, along with no significant differences in all-cause and cardiac death. These findings are more reliable in ACS or low-risk patients with stable angina, where the benefit from reduced bleeding is necessary. The incidence of any other bleeding was 1.25% lower with shorter-term DAPT compared to a 2.02% risk with standard-term DAPT, resulting in an absolute risk reduction of 0.77%. There was a significant absolute risk reduction of 0.25% in major bleeding in the short DAPT group (0.32% versus 0.57%) [18].

The rebound effect demonstrated in the DAPT trial refers to the occurrence of ischemic events, such as MI or ST, shortly within three months after discontinuation of the P2Y12 inhibitor in DAPT. This rebound phenomenon was observed whether the cessation of DAPT was done at 12 months or after 30 months, as seen in the DAPT trial. The PEGASUS-TIMI 54 trial addressed this issue by comparing DAPT, consisting of aspirin and ticagrelor, and the other group with aspirin alone. The trial found that continuing DAPT after a brief interruption (≤30 days) of the P2Y12 inhibitor resulted in more pronounced effects for secondary prevention of ischemic events. However, the study did not provide data on DAPT discontinued before the one-year period [20]. Other trials, such as RESET and OPTIMIZE, compared the effects of three-month versus 12-month DAPT regimens, while EXCELLENT, PRODIGY, SECURITY, and ITALIC trials evaluated six-months versus 12- or 24-month DAPT durations [20]. Table 12 outlines the nonsignificant risk of ST associated with short and long-term DAPT [20].

**Table 12:** DAPT duration w.r.t the type of ST DAPT=dual anti-platelet therapy, ST=stent thrombosis, MI=myocardial infarction Adapted from Piccolo et al. [20]

Endpoint	Short DAPT (≤6 months) (n = 5,730)	Long DAPT (12 months) (n = 5,743)	HR (95% CI)	p-value
MI or ST	15 (0.27%)	16 (0.29%)	0.93 (0.46–1.90)	0.85
Definite ST	5 (0.09%)	2 (0.04%)	2.48 (0.48–12.74)	0.28
Definite or probable ST	6 (0.11%)	3 (0.05%)	1.98 (0.50–7.92)	0.33

To compare the early adverse events after discontinuation of DAPT, two groups were examined: one discontinuing DAPT within one year (short term) and the other discontinuing it after 1 year of use (long term). Both groups had received DES. There was no significant difference in the occurrence of ST or MI, but MACE was higher in the group discontinuing DAPT after long-term use. Subgroup analysis conducted 90 days before and 90 days after DAPT discontinuation showed a significant reduction in bleeding risk (p=0.022), which was more favorable in the short-term DAPT group [20]. The PRECISE-DAPT score is a scoring system used to evaluate DAPT duration based on the assessment of predicting factors, primarily focusing on out-of-hospital bleeding risk. Table 13 outlines the components of the PRECISE-DAPT scoring system [8].

**Table 13:** PRECISE-DAPT score to determine DAPT duration w.r.t bleeding risk WBC=white blood cells, Cr=Creatinine, DAPT=dual anti-platelet therapy, PRECISE-DAPT=Predicting Bleeding Complication in Patients Undergoing Stent Implantation and Subsequent Dual Antiplatelet Therapy Score <25-no high risk bleeding=standard DAPT Score >25-high risk bleeding-short term DAPT Adapted from Valgimigli et al. [8]

PRECISE-DAPT score	
Hemoglobin (1g/dl decrease)	0-15
WBC (10^3^ units/^µl^ increase)	0-15
Age (10 years increase)	0-19
Cr clearance (10ml/min decrease)	0-25
Prior bleeding	0-26
Total	0-100

Similarly, nine predicting factors have been formulated to guide risk scoring for deciding DAPT duration beyond 12 months with scores ranging from -2 to +10. In the DAPT trial, high-risk-scored patients (i.e. a score ≥2) continued DAPT to show a reduction in MI/ST and cardiovascular and cerebrovascular event risk with 30 months of DAPT, along with a modest increase in bleeding. Low-risk-scored patients (i.e., score <2) did not show a reduction in ischemic events with prolonged DAPT but had a significant increase in moderate and major bleeding risk, suggesting to stop DAPT continuation beyond 12 months. Table 14 outlines the factors used to calculate the DAPT score [8].

**Table 14:** DAPT score system for guiding duration of anti-platelet therapy DAPT=dual anti-platelet therapy, CHF=congestive heart failure, LVEF=left ventricular ejection fraction, MI=myocardial infarction, PCI=percutaneous coronary intervention, PES=paclitaxel-eluting stent DAPT score ranges from -2 to +10 with different predicting factors influenced by their specific score value. Adapted from Valgimigli et al. [8]

DAPT scoring	
Age	
>75 years	-2 points
65-<75years	-1 points
<65 years	0 points
CHF/low LVEF	2 points
Vein graft stenting	2 points
MI at presentation	1 point
Prior MI or PCI	1 point
Diabetes	1 point
Stent diameter <3 mm	1 point
Smoking	1 point
Paclitaxel-eluting stent	1 point

Among the different DAPT options, prasugrel has been shown to have faster and stronger platelet inhibition compared to clopidogrel. The TL-PAS trial investigated the use of prasugrel plus aspirin for 12 months following stent placement and demonstrated a significant reduction in the incidence of MI (1.9% vs. 7.1%; HR 0.255; P < 0.001), as well as a reduction in ST (0.2% vs. 2.9%; HR 0.063; P < 0.001). In the GUSTO study, although severe bleeding was numerically higher in patients continuing prasugrel for 30 months, the results were not statistically significant. It is worth noting that older age (≥75 years), body weight <60 kg, and previous stroke or transient ischemic attack (TIA) were identified as factors that made certain populations more susceptible to adverse events with prasugrel compared to clopidogrel [8].

Ticagrelor is another novel oral drug that reversibly binds to and inhibits the P2Y12 receptor. In the PLATO trial, a cohort study, ticagrelor was compared to clopidogrel and given to patients undergoing PCI for ACS for a duration of 12 months. The ticagrelor group showed a clear superiority over clopidogrel, with lower incidence of ST (1.3% vs. 1.9%; P < 0.01), reduced risk of total mortality (4.5% vs. 5.9%; P < 0.001), and decreased rates of death from vascular causes and MI (HR 0.84, 95% CI 0.77-0.92; P < 0.001). No significant difference in bleeding risk was observed between the two groups [8].

Recent studies have investigated the concomitant use of clopidogrel and PPIs (proton pump inhibitors) in patients at higher risk of gastrointestinal injury. The results indicate a significantly higher risk of adverse cardiovascular events, including MACEs, ST, and MI, following PCI, particularly with the use of omeprazole. This association can be explained by the fact that both drugs are metabolized by the same enzyme, mainly the CYP2C19 isoenzyme. The comparative results after one year demonstrated a lower risk of ST in patients who did not use PPIs (OR with 95% CI = 1.38 (1.13-1.70); p-value = 0.002). The randomized PRODIGY trial focused on patients using non-omeprazole PPIs and suggested that these types of PPIs might be safer to use in combination with clopidogrel. During the long-term follow-up period, patients using clopidogrel alone had significantly better outcomes in terms of MACE, MI, and ST. However, the studies that reviewed this analysis did not mention any specific association of complications with a particular stent type, whether BMS or DES. Further research is needed to provide recommendations regarding individual PPIs [21].

P2Y12 antagonist monotherapy following a short duration of three months of DAPT with aspirin may be non-inferior to the standard DAPT duration in reducing aspirin-related adverse effects, such as bleeding, which otherwise has a significant 40% chance of major bleeding risk occurrence. Bleeding can interrupt antiplatelet function, predisposing patients to thrombotic risks. The SMART-CHOICE trial and the STOPDAPT-2 trial enrolled low-risk patients and supported the use of monotherapy, showing a decreased risk of ischemic events (MI, ST, stroke). The TWILIGHT study focused on high-risk patients undergoing PCI and also demonstrated a 51% risk reduction in bleeding. A pooled analysis of RCTs showed nonsignificant differences in ST between DAPT and monotherapy groups (0.5% for DAPT vs. 0.4% for monotherapy; RR, 1.14; 95% CI, 0.81-1.61; P = 0.44). However, the available data in this area is limited, and further RCTs are necessary to confirm and establish these findings [22].

The efficacy of bivalirudin versus unfractionated heparin (UFH) plus glycoprotein IIb/IIIa inhibitor in STEMI patients undergoing primary PCI was studied in the HORIZON-AMI trial. The results showed a reduction in major bleeding, thrombocytopenia, and lower mortality rates at 30 days and three years, with bivalirudin. However, there was a 1.3% higher rate of acute ST within the first 24 hours in the group receiving bivalirudin as a short infusion. STEMI patients have high platelet activity predisposing to acute ST. Also, the rapid clearance of bivalirudin after discontinuation provides insufficient antithrombotic protection until oral antiplatelet activity takes effect. This suggests the need for a rapid-acting intravenous antiplatelet during the transition period to bridge the gap in antithrombotic protection [11].

Cangrelor is an intravenous antiplatelet with a P2Y12 receptor inhibitor. It was studied in the CHAMPION PHOENIX trial, comparing it to clopidogrel for the reduction of periprocedural ischemic events in patients undergoing PCI on a background of bivalirudin (a direct thrombin inhibitor anticoagulant). Compared to clopidogrel, cangrelor demonstrated a relative reduction in ST (48 (4.7%) vs. 70 (6.7%); odds ratio (OR): 0.68, p = 0.047) within 48 hours, with a trend towards decreased ST becoming evident within two hours after PCI (p = 0.057). GUSTO minor bleeding risk (0.2%) and AQUITY-defined major bleeding were reported, although both scoring systems deemed it nonsignificant. Here, bivalirudin could act as a countermeasure to mitigate the bleeding risk that may arise when cangrelor is used. The rescue of glycoprotein IIb/IIIa inhibitors was also lower with cangrelor compared to clopidogrel (1.4% vs. 3.1%, OR: 0.44 (95% CI: 0.24 to 0.84); p = 0.010) [11].

Although trials comparing different dosages and infusion durations of bivalirudin with unfractionated heparin (UFH) did not reach a conclusive superiority of bivalirudin in bleeding prevention, some specific trials offer valuable insights. For instance, the EUROMAX trial focused on STEMI patients with a higher risk of ST and used a low dose of bivalirudin (0.25 mg/kg per hour). It found a significantly higher rate of definite ST within 24 hours in the bivalirudin group compared to the UFH group (1.1% vs. 0.2%, p = 0.007). However, a post-hoc analysis of EUROMAX showed reduced incidence of ST with an extended three-hour duration of bivalirudin infusion at the PCI dose (1.75 mg/kg per hour), compared to that with UFH (0.5% vs. 0.2%; p = 0.45) [11].

On the other hand, the HEAT-PPCI trial which used the newer P2Y12 receptor antagonists ticagrelor or prasugrel by 89% and focused on participants with a high coronary artery thrombus burden, identified that bivalirudin infused at the end of the procedure had a higher but similar positive incidence of definite ST at 28 days compared to the UFH group (3.4% vs. 0.9%, p = 0.001) [11].

Despite the higher early ST rate, the European Society of Cardiology guidelines recommend the use of bivalirudin over UFH for patients undergoing primary PCI due to the observed mortality benefit with reduced major bleeding risk in bivalirudin-treated patients during the subacute period (1-30 days). Conversely, the American Heart Association guidelines suggest that either drug can be used for patients undergoing primary angioplasty [11].

Limitations

This study did not include articles discussing different generations of DES, each with their comparative efficacy in detail. Articles determining the relationship between specified comorbidities and their underlying pathogenesis in the development of ST were excluded. Articles investigating multi-vessel disease and their association with the development of ST were not chosen for this article.

## Conclusions

ST is an uncommon but feared complication after PCI, especially in patients with ACS. Although timely intervention and efficient DES usage, second-generation DES in particular have played a significant role in the reduction of ST. Optimal intravascular imaging during stenting and post-procedure DAPT has played a game-changing effect with a drastic decrease in ST incidence and other subsequent complications. Nonetheless, the recent ISCHEMIA (International Study of Comparative Health Effectiveness with Medical and Invasive Approaches) trial, demonstrated that patients with early invasive intervention did not differ from the group of patient population receiving conservative medical treatment, in terms of the risk of ischemic events in CAD patients with moderate to severe ischemic heart failure. It gives us an insight into the importance of patient and lesion-related factors, mandating patient education for optimizing lifestyle changes. Hence, all measures should be taken into account to achieve state-of-the-art results from PCI.
